# Evaluation of Quality of Record-Keeping and Root Canal Therapy Performed by Two Predoctoral Cohorts

**DOI:** 10.3390/dj13040174

**Published:** 2025-04-19

**Authors:** Wei Chun Yeoh, Chun Giok Koay, Genevieve Yuiin Sze Kong, Emilyn Wenqi Gan, Rikan Libat, Muneer Gohar Babar, Abhishek Parolia

**Affiliations:** 1 School of Dentistry, International Medical University, Kuala Lumpur 57000, Malaysia; yeoh.weichun@student.imu.edu.my (W.C.Y.); koay.chungiok@student.imu.edu.my (C.G.K.); genevieve.kongyuiin@student.imu.edu.my (G.Y.S.K.); emilyn.ganwen@student.imu.edu.my (E.W.G.); rikan.libat@student.imu.edu.my (R.L.); muneer_babar@imu.edu.my (M.G.B.); 2 Department of Endodontics, College of Dentistry and Dental Clinics, University of Iowa, Iowa City, IA 52242, USA

**Keywords:** clinical audit, quality of record-keeping, root canal therapy

## Abstract

**Objectives:** A retrospective clinical audit was carried out to evaluate and compare the quality of record-keeping (QRK) and quality of root canal therapy (QRCT) performed by 4th-year and 5th-year predoctoral students. **Methods:** Electronic records and periapical radiographs of 702 root canal treated teeth performed by 4th-year and 5th-year predoctoral students from July 2018 to December 2021 were evaluated in compliance with standard European Society of Endodontology (ESE) and American Association of Endodontists (AAE) guidelines. Associations between the QRK and the QRCT were statistically analysed using the chi-square test (*p* < 0.05). **Results:** Overall acceptability of the QRK and the QRCT was 72.08% and 50.57%, respectively. The reference point of working length was the most common criterion not recorded (33.91%). No significant difference was observed in the QRK between 4th-year (76.05%) and 5th-year (69.70%) students (*p* = 0.226), as well as the QRCT between 4th-year (51.33%) and 5th-year (50.11%) students (*p* = 0.755). Acceptable root canal fillings were significantly higher in anterior teeth (57.48%) than in posterior teeth (47.54%) (*p* = 0.015). Satisfactory QRK was significantly associated with satisfactory QRCT (*p* = 0.046). **Conclusions:** Both predoctoral cohorts showed no difference in QRK and QRCT. However, QRK was better than QRCT. Comprehensive and accurate record-keeping positively impacted the QRCT.

## 1. Introduction

Root canal therapy (RCT) is a cornerstone intervention in the management of endodontic pathology, designed to eliminate microbial colonization within the root canal system and prevent reinfection through comprehensive chemo-mechanical debridement followed by three-dimensional obturation [1,2]. The long-term success of endodontic therapy is contingent on various factors, among which the quality of root canal fillings plays a pivotal role in determining treatment prognosis [3]. Poorly executed RCT, as documented in multiple epidemiological studies, has frequently been correlated with persistent periapical pathosis, underlining the clinical consequences of suboptimal endodontic practice [4,5,6].

In parallel, the quality of clinical record-keeping has garnered increasing recognition within healthcare, particularly in its role in ensuring the continuity of care, facilitating treatment audits, and supporting medicolegal accountability. Endodontic therapy, which often involves multi-step procedures over multiple appointments, necessitates precise and detailed documentation. Accurate records not only enable seamless treatment progression but also mitigate procedural errors, such as omissions or misinterpretations, which could compromise treatment outcomes. Furthermore, meticulous documentation serves as a robust safeguard in medicolegal situations, outlining potential procedural complications and ensuring legal accountability [7].

Despite the established importance of both technical excellence in RCT and high-quality record-keeping, there is a paucity of literature exploring their potential interrelationship. While previous studies have extensively analysed technical factors affecting endodontic success [8,9] and demonstrated that structured documentation training enhances compliance and completeness in dental education [10,11], the impact of meticulous record-keeping on procedural outcomes in endodontics remains underexplored [12]. In educational settings, where students rely on structured records for guidance and faculty feedback, the role of documentation in reinforcing treatment standards is particularly relevant.

To address this gap, this study investigates how clinical competence in endodontics evolves by comparing 4th- and 5th-year predoctoral students in terms of both the quality of root canal therapy (QRCT) and the quality of record-keeping (QRK). Fourth-year students, as they transition from preclinical simulations to supervised patient care, are expected to establish foundational technical skills while adhering to structured documentation protocols. In contrast, 5th-year students, having accumulated greater clinical exposure and procedural autonomy, may demonstrate enhanced technical proficiency but potentially variable adherence to documentation standards. Prior research has explored the influence of seniority on QRCT, often reporting that increased experience correlates with improved technical performance [13,14]. However, its impact on QRK remains insufficiently examined.

This study systematically assesses whether superior documentation practices correlate with enhanced technical performance in RCT, providing insights into how clinical record-keeping and endodontic proficiency evolve across different training levels. Understanding this relationship could inform curricular advancements in predoctoral education, reinforcing structured documentation as an integral component of clinical excellence and quality assurance in endodontic training.

## 2. Material and Methods

### 2.1. Preclinical Training of Predoctoral Students

Predoctoral students underwent extensive preclinical instruction in RCT using extracted human anterior and posterior teeth as models. Prior to engaging in patient care, predoctoral students were required to successfully complete a structured preclinical competency assessment during their third year. This assessment required them to perform a full RCT, including access cavity preparation, working length determination, master cone selection, obturation, and temporization, on both a single-canalled tooth and a molar. Each critical step was rigorously evaluated by the examiners to ensure technical proficiency. The students were given two 150 min sessions to complete the procedure on the designated tooth. Furthermore, as part of their final-year clinical competency, students must perform a complete RCT on a single-canalled tooth in a clinical environment, demonstrating the translation of their preclinical skills to real patient care.

### 2.2. RCT Protocol

Case complexity was standardized using the American Association of Endodontists (AAE) Endodontic Case Difficulty Assessment Form to ensure consistency in treatment difficulty across student cohorts [15]. Only cases classified as minimal or moderate difficulty, while remaining within the scope of predoctoral competency, were allocated to predoctoral students.

All root canal procedures adhered to strict rubber dam isolation protocols. Utilizing endodontic access burs (Dentsply Maillefer, Ballaigues, Switzerland), access cavities were prepared. Electronic apex locators (Root ZX II, J Morita, Kyoto, Japan) were used to locate the root apices, as confirmed radiographically. Shaping and cleaning procedures involved hand K-files and hand Protaper files (Dentsply Maillefer, Ballaigues, Switzerland), and irrigations with 2% sodium hypochlorite (NaOCl) (Clorox Company, Broadway, Oakland, CA, USA), saline, and 17% ethylene diamine tetra acetic acid (EDTA) (Ultradent Products Inc., South Jordan, UT, USA).

Non-setting calcium hydroxide intracanal dressing (Calcicur, Voco Gmbh, Cuxhaven, Germany) was employed between visits. Temporary restorative materials, such as reinforced zinc oxide (IRM, Dentsply Caulk, Milford, DE, USA) or Cavit (3M ESPE, St. Paul, MN, USA), were used between appointments to ensure an effective coronal seal.

In the final visit, root canals were obturated with gutta-percha cones coated with AH Plus (Dentsply Maillefer, Ballaigues, Switzerland) sealer using the lateral compaction technique. Subsequently, root canals were sealed with flowable composite resin (Beautifil Injectable X, Shofu, Japan), followed by packable composite resin (Beautifil II, Shofu, Japan) or post and core (Rely X Fibre Post 3D, Two Harbours, MN, USA) after an assessment of the remaining tooth structure.

### 2.3. Data Collection

Commencing with the approval from the institutional ethical committee and patients’ consent, the electronic records of RCT performed by 4th-year and 5th-year predoctoral students at Oral Health Clinic, Kuala Lumpur, Malaysia, from July 2018 to December 2021 were retrieved from Open Dental Software, version 22.3 (Open Dental, Salem, OR, USA). Records of ongoing or incomplete endodontic treatment within this clinical audit timeframe, as well as poor-quality radiographs with artefacts, were excluded.

### 2.4. Training and Calibration of Examiners

Five examiners received supervision and training by an endodontist with over 20 years of expertise in micro-endodontics as followed to ensure reliability and consistency in gathering the data:

- A two-hour lecture and discussion on the principles and methodologies of a clinical audit;
- Discussion of the literature review on the evaluation criteria for the QRK and QRCT;
- Development of a checklist to assess the QRK and QRCT.

A calibration phase was executed during which the examiners and endodontist independently evaluated 10 patients in a pilot study. Cohen’s Kappa test was used to assess each examiner’s reliability, and an inter-examiner agreement of 0.85 Cohen Kappa value was achieved.

### 2.5. Quality of Record-Keeping

Examiners independently assessed the QRK in the electronic records against a criteria checklist developed in accordance with the European Society of Endodontology (ESE) and AAE guidelines [16,17]. The criteria documented in the electronic records were used to assess the QRK (Table 1). QRK was classified as good if all the criteria were comprehensively documented.

### 2.6. Quality of RCT

Pre-operative, working length determination, master cone, and post-operative radiographs of all root canal treated teeth were evaluated. Examiners independently assessed the QRCT based on the condensation, extension, and the absence or presence of procedural mishaps using full-screen computer images, conforming to the ESE and AAE guidelines [16,17] (Table 2). In multirooted teeth, each root canal was individually assessed, and a cumulative score was assigned to the tooth. The overall QRCT was classified as acceptable only if all canals met the predefined criteria.

### 2.7. Data Analysis

Collected data were tabulated in Microsoft Excel 2016 (Microsoft, Redmond, WA, USA). Utilizing SPSS software version 18.0 (SPSS, Inc., Chicago, IL, USA), distributions of the QRK and QRCT were calculated. Factors associated with QRCT, including QRK, tooth type, and student seniority, were assessed using Pearson chi-square analysis, with the level of significance set at *p*-value < 0.05.

## 3. Results

A total of 702 root-filled teeth were evaluated, comprising 296 male patients (42.17%) and 406 female patients (57.83%). The middle-aged adult group (>50 years old) accounted for the highest proportion of treated patients at 38.18% (*n* = 268). Among the assessed root-filled teeth, premolars were the most frequently treated (41.03%, *n* = 288), followed by molars (28.49%, *n* = 200), incisors (23.36%, *n* = 164), and canines (7.12%, *n* = 50).

The overall acceptability of QRCT was 50.57% (*n* = 355) (Table 3). Among the 702 teeth examined, 544 (77.49%) demonstrated acceptable condensation of root canal fillings, 561 (79.91%) had acceptable extension, and 581 (82.76%) exhibited no procedural mishaps (Table 3).

**Figure 1:**
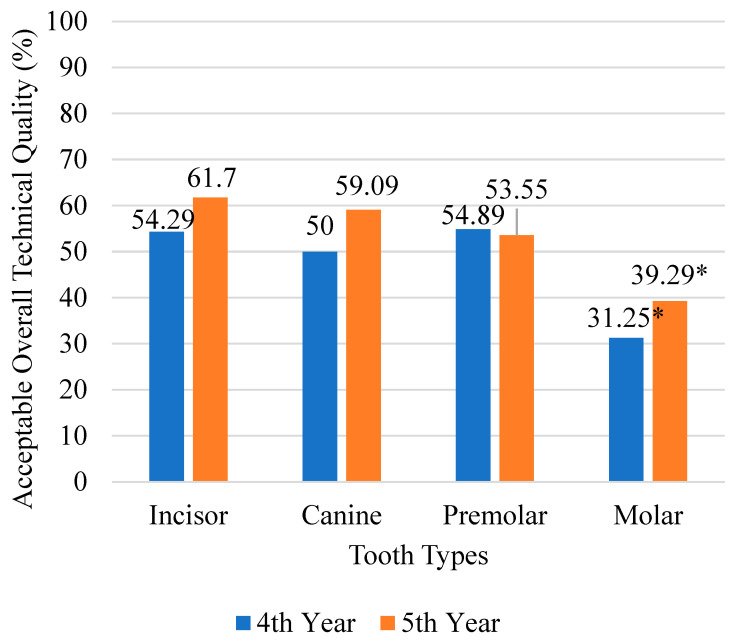
Assessment of the technical quality acceptability of root canal fillings concerning tooth type and student seniority. * *p* < 0.05.

A significant difference was observed in QRCT acceptability across tooth types, with molars exhibiting the lowest acceptability in condensation (*n* = 133, 66.50%) and extension (*n* = 138, 69.00%) compared to other teeth (*p* < 0.001) (Table 4). Anterior teeth (*n* = 123, 57.48%) demonstrated significantly higher acceptability of root canal fillings than posterior teeth (*n* = 232, 47.54%) (*p* = 0.015) (Table 4). Molars showed the lowest QRCT acceptability in both 4th-year (*n* = 10, 31.25%) and 5th-year predoctoral students (*n* = 66, 39.29%) (*p* = 0.016) (Figure 1).

Procedural mishaps were significantly more frequent in molars (*n* = 48, 24.00%) compared to other tooth types (*p* = 0.021) (Table 4). However, no significant difference was observed in the overall QRCT between 4th-year (*n* = 135, 51.33%,) and 5th-year predoctoral students (*n* = 220, 50.11%) (*p* = 0.755) (Table 4).

**Figure 2:**
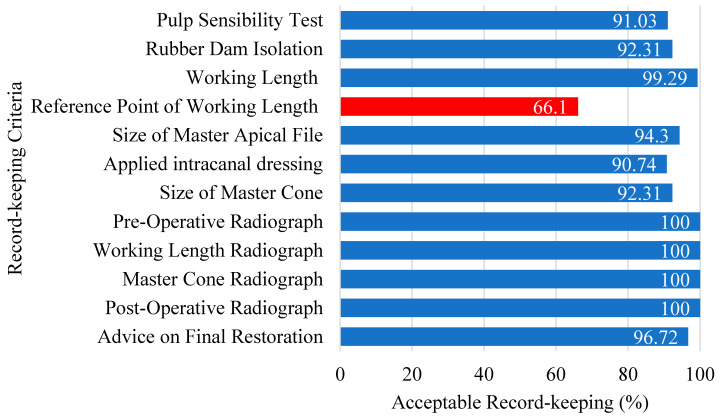
Compliance with record-keeping guidelines.

The overall acceptability of record-keeping was 72.08% (*n* = 506), with 4th-year students demonstrating 76.05% (*n* = 200) acceptability and 5th-year students 69.70% (*n* = 306) 69.70% (Table 4). No significant association was found between QRK acceptability and student seniority (*p* = 0.226). The coronal reference point of the working length was the most frequently omitted criterion, with only two-thirds of records classified as acceptable (*n* = 464, 66.10%) (Figure 2). The QRK was deemed to be significantly associated with the QRCT (*p* = 0.046). Satisfactory record-keeping was positively correlated to satisfactory technical quality of root canal fillings.

## 4. Discussion

Numerous studies have delved into the technical proficiency of root canal fillings executed by predoctoral students, showcasing diverse outcomes of the adequacy of the root fillings [18,19,20]. Ribeiro et al. reported a high prevalence of endodontic errors, including ledge formation, furcal and apical perforations, in RCTs performed by predoctoral students [21]. In contrast to their meta-analysis findings, the present study identified the absence of procedural mishaps as the most frequently met criterion for satisfactory RCT outcomes. However, the homogeneity of root canal fillings emerged as the least satisfactory technical criterion in the present study, consistent with observations by Peters, Sonntag and Peters, which attributed suboptimal obturation to inadequate gutta-percha compaction in the lateral compaction technique, canals of irregular anatomy, and discrepancies in spreader penetration and cone fit [22]. Similarly, El-Ma’aita et al. highlighted the underextension of root canal obturation as a prevalent endodontic error among undergraduates [23]. Discrepancies across studies may stem from variations in evaluation criteria, sample sizes, examiner calibration, teaching methods, sample sizes, and differences in the clinical experience of predoctoral students across institutions worldwide.

Consistent with the findings by Balto et al., Barrieshi-Nusair et al., and Vukadinov, molars exhibited a significantly higher occurrence of unacceptable root canal fillings compared to other tooth types [24,25,26]. The acceptability of condensation and extension in molar root canal fillings was markedly lower than in anterior and premolar teeth, in line with the conclusions drawn by Khabbaz, Yavari, Moussa-badran [18,27,28]. The higher prevalence of procedural mishaps in molars can largely be attributed to their complex internal morphology, which includes narrow, curved canals and multiple root configurations, posing significant challenges in negotiation, instrumentation, and obturation. These anatomical constraints, compounded by reduced accessibility and visibility in the posterior region, contribute to the difficulty in achieving optimal obturation quality [29,30]. Beyond anatomical factors, student-related variables may further impact QRCT outcomes in molars. Prior studies by Baaij et al. and Puryer et al. have highlighted diminished self-perceived confidence among predoctoral students when performing molar endodontics, which may contribute to hesitancy, suboptimal decision-making, and, ultimately, poorer technical outcomes [31,32]. A discernible increase in procedural errors was observed in molars treated by 4th-year predoctoral students when juxtaposed with those treated by their 5th-year counterparts. This discrepancy in performance may be ascribed to the 4th-year cohort’s comparatively nascent proficiency and limited exposure to the intricacies of multirooted dental structures.

Conversely, their more seasoned 5th-year peers, having undergone greater clinical training and refinement of motor skills, demonstrate a more adept approach to molar endodontics. However, despite their additional training, 5th-year students did not achieve significantly superior QRCT outcomes than their 4th-year counterparts when treating molars. This suggests that clinical experience alone may not be sufficient to overcome the inherent anatomical challenges of molar endodontics. The lack of a significant difference in QRCT outcomes between student cohorts indicates that while predoctoral training adequately prepares students for straightforward cases, it may not fully equip them to manage the complexities of molar endodontics with consistency. This highlights the need for structured training interventions, such as targeted workshops, case-based learning, and faculty-guided clinical simulations, to bridge the gap between general clinical experience and the advanced skill set required for predictable molar RCT outcomes [33]. The incorporation of rotary instrumentation in undergraduate curricula has been shown to improve QRCT, by facilitating efficient canal preparation, minimizing procedural errors, and preserving root canal anatomy [34]. Close supervision by endodontists, and strategic allocation of cases based on seniority, can improve the QRCT [35,36]. Additionally, the integration of magnification tools such as loupes with lights and dental operating microscopes (DOM) and exposure to rotary instrumentation during undergraduate training may enhance visualization, improve technical precision, and optimize QRCT outcomes in molar cases [34,37,38].

This study demonstrated a commendable overall acceptability of record-keeping in endodontic treatment among predoctoral students. While the findings indicated that 4th-year predoctoral students exhibited superior QRK compared to their 5th-year counterparts, there was no significant association between student seniority and record-keeping. Several factors may explain why 4th-year students showcased better documentation practices despite their comparatively lower clinical experience. First, differences in cognitive load and prioritization may contribute to this trend. While 4th-year students are still developing their technical proficiency, they may place greater emphasis on adhering to structured assessment criteria that prioritize documentation. In contrast, 5th-year students, who have gained greater procedural confidence, may prioritize technical execution over detailed record-keeping. The increasing complexity of cases at this stage may further divert their attention away from documentation, leading to omissions of non-mandatory details. Second, compliance with formal assessment criteria may influence documentation behaviour. Fourth-year students, being newly introduced to clinical record-keeping evaluations, may be more diligent in adhering to structured documentation protocols. Conversely, 5th-year students, accustomed to clinical workflows, may demonstrate more selective compliance, focusing on essential but not necessarily exhaustive documentation.

The coronal reference point of the working length was the most frequently omitted criterion in record-keeping. Inadequate documentation of this crucial reference point may lead to the loss of vital information, potentially resulting in underfilling or overfilling of the root canal, thus compromising the QRCT and leading to unsatisfactory endodontic outcomes. However, the absence of a recorded coronal reference point did not significantly correlate with unacceptable extension. This suggests that the omission of documentation does not necessarily reflect a neglect of the concept and application of the coronal reference point by predoctoral students during clinical practice. Pourasghar et al. proposed that the omission of information in medical records may arise from students or clinicians prioritizing certain treatment steps over others, potentially at the expense of documentation completeness [39].

A significant positive correlation was observed between QRK and QRCT. This suggests that deficient documentation practices among predoctoral students affect the quality of root canal obturation due to the loss of critical procedural information. Considering the multifaceted nature of endodontic treatments, necessitating multiple appointments and comprising various intricate procedures, the omission of key information, such as working length, coronal reference point, and master cone size, can hinder procedural accuracy and compromise treatment outcomes. Several mechanisms may explain this relationship. First, structured documentation reinforces adherence to clinical protocols, ensuring that procedural steps—such as accurate working length determination and obturation technique—are systematically recorded and executed. Second, comprehensive records facilitate faculty supervision and targeted feedback, allowing instructors to identify and correct errors more effectively [40]. In contrast, incomplete documentation may obscure procedural deficiencies, limiting opportunities for corrective guidance and skill refinement. Furthermore, high-quality QRK may reflect a more disciplined clinical approach, wherein students who maintain meticulous records also demonstrate greater attention to detail and procedural accuracy. Amos et al. postulated that structured record-keeping fosters a systematic approach to clinical training, reinforcing both technical competence and professional responsibility [11].

Inadequate record-keeping poses significant risks for predoctoral students, potentially hindering their ability to defend against allegations of clinical negligence or professional misconduct. Rios Santos et al. emphasized that dental schools play a pivotal role in mitigating these risks and shaping future practitioner behaviour [41]. A proactive strategy to address this issue is the implementation of structured endodontic record-keeping templates, which would prompt predoctoral students to systematically document essential procedural details [42]. The academic curriculum should integrate dedicated training sessions on record-keeping to educate students on the importance of comprehensive documentation in clinical practice. During preclinical lectures and clinical sessions, the faculty should emphasize the significance of each criterion in endodontic record-keeping, reinforcing its role in treatment continuity, patient safety, and medicolegal protection. Clinical supervisors should actively review student documentation for each case, providing constructive feedback to reinforce best practices and ensure adherence to standardized documentation protocols.

### Strengths and Limitations

The present study introduces a novel approach by evaluating the QRK among predoctoral students and its correlation with the QRCT. The authors advocate for the inclusion of comprehensive pre- and post-operative assessments of oral health-related quality of life (OHRQoL) as a critical component of standardized record-keeping protocols in contemporary endodontics. Incorporating this metric would provide clinicians with valuable baseline data, facilitating further exploration of the interplay between QRK and QRCT, while also offering insights into their broader implications on patients’ overall quality of life (QoL).

A primary limitation of this study is its reliance on periapical radiographs for assessing QRCT. Although widely used in endodontic assessments, their two-dimensional nature restricts the ability to fully visualize the complexity of the root canal system, thus limiting the accurate depiction of the quality of disinfection of the root canal system. Superimposition of anatomical structures further complicates radiographic interpretation. Not all students effectively applied the parallax radiographic technique in multirooted teeth, impeding the accurate evaluation of the condensation and length of root canal fillings [43].

Additionally, the retrospective nature of this study inherently limits the ability to establish causal relationships between QRK and QRCT. Given that data were collected from pre-existing clinical records, potential inconsistencies in documentation and variations in faculty supervision may have influenced the findings. Differences in faculty oversight, frequency of feedback, and intervention levels could have impacted students’ adherence to procedural protocols. Future studies should consider stratifying cases based on supervision intensity to assess its direct impact on QRCT performance.

Selection bias is another consideration, as the study exclusively analysed cases completed by predoctoral students under specific clinical settings, which may not be fully generalizable to other dental institutions or private practice settings. Future studies with a prospective design and the integration of three-dimensional imaging modalities, such as cone-beam computed tomography (CBCT), may offer a more comprehensive assessment of QRCT and its correlation with QRK.

## 5. Conclusions

QRK and QRCT were acceptable in at least half of the cases performed by predoctoral students. Comprehensive and meticulous documentation in endodontics is critical in enhancing QRCT outcomes. The structured development of standardized record-keeping templates, based on evidence-based documentation guidelines, along with routine faculty-led reviews of clinical records and formative feedback, should be integrated into the curriculum to enhance procedural accuracy, reinforce clinical accountability, and cultivate a systematic approach to endodontic practice among predoctoral students.

## Figures and Tables

**Table 1:** Criteria used to assess the quality of record-keeping.

Use of pulp sensibility and other tests to formulate pulpal and periapical diagnosis
2. Use of local anaesthesia
3. Name of local anaesthesia
4. Dosage of local anaesthesia
5. Use of rubber dam isolation
6. Working length
7. Reference point of working length
8. Size of initial apical file
9. Size of master apical file
10. Applied intracanal dressing
11. Medication prescribed including analgesics and antibiotics
12. Size of master cone
13. Pre-operative radiograph
14. Working length radiograph
15. Master cone radiograph
16. Post-operative radiograph
17. Advice on final restoration in the follow-up visit

**Table 2:** Criteria utilized to evaluate the quality of root canal therapy.

Variables	Criteria	Definition
Condensation	Acceptable	Absence of voids within the root canal fillings or at the interface between the filling material and root canal walls
	Non-acceptable	Detection of voids within the root canal fillings or at the interface between the filling material and root canal walls
Extent	Acceptable	Root canal filling material is confined within the root canal system and extends up to 2 mm from the radiographic apex
	Underfilled	Root canal filling material terminates more than 2 mm short of the radiographic apex
	Overfilled	Overextension of root canal filling material beyond the radiographic apex
Mishap	Absence of mishap	No mishap identified
	Gouging	Overextension of the access cavity undermining the enamel walls as apparent by radiograph
	Ledge	An artificial irregularity created on the surface of the root canal wall
	Perforation	Iatrogenic communication between root canal system and the external tooth surface
	Transportation	Post-instrumentation root canal walls were not the result of uniform preparation of the initial root canal walls
	Separation of instruments	Separated instrument identified
	Zipping	Transposition of the apical portion of the canal
Overall	Acceptable	Root canal fillings exhibit adequate condensation and optimal length, with no procedural mishaps
	Non-acceptable	Root canal fillings display inadequate condensation and/or suboptimal length, with or without procedural mishaps

**Table 3:** Quality classification of root canal fillings performed by predoctoral students: acceptable versus non-acceptable.

Variables	Criteria	Total
Condensation	Acceptable	544 (77.49%)
	Non-acceptable	158 (22.51%)
Extent	Acceptable	561 (79.91%)
	Underfilled	90 (12.82%)
	Overfilled	51 (7.26%)
Mishap	Absence of mishap	581 (82.76%)
	Gouging	47 (6.70%)
	Ledge	29 (4.13%)
	Perforation	22 (3.13%)
	Transportation	15 (2.14%)
	Separation of instruments	6 (0.85%)
	Zipping	3 (0.43%)
Overall	Acceptable	355 (50.57%)
	Non-acceptable	347 (49.43%)

**Table 4:** Analysis of acceptable root canal fillings in relation to tooth type, student seniority, and record-keeping quality.

		Total	Total of Overall Acceptable	Acceptable Condensation	Acceptable Extension	Mishap
Tooth type	Incisors	164	96 (58.54%)	126 (76.83%)	146 (89.02%)	27 (16.46%)
	Canines	50	27 (54.00%)	35 (70.00%)	40 (80.00%)	6 (12.00%)
	Premolars	288	156 (54.17%)	250 (86.81%)	237 (82.29%)	40 (13.89%)
	Molars	200	76 (38.00%) **	133 (66.50%) *	138 (69.00%) *	48 (24.00%) *
Student seniority	4th	263	135 (51.33%)	193 (73.38%)	214 (81.39%)	50 (19.01%)
	5th	439	220 (50.11%)	351 (79.95%)	347 (79.04%)	71 (16.17%)
Quality of record-keeping	Good †		506 (72.08%)			
	Poor †		196 (27.92%)			
Acceptable quality of record-keeping	4th 5th	263 439	200 (76.05%) 306 (69.70%)			

* *p* < 0.01. ** *p* < 0.05. † High-quality record-keeping (11–17 criteria documented); low-quality record-keeping (fewer than 11 criteria documented).

## Data Availability

The data supporting the findings of this study can be obtained from the corresponding author upon reasonable request.
